# Almond diversity and homozygosity define structure, kinship, inbreeding, and linkage disequilibrium in cultivated germplasm, and reveal genomic associations with nut and seed weight

**DOI:** 10.1038/s41438-020-00447-1

**Published:** 2021-01-10

**Authors:** Stefano Pavan, Chiara Delvento, Rosa Mazzeo, Francesca Ricciardi, Pasquale Losciale, Liliana Gaeta, Nunzio D’Agostino, Francesca Taranto, Raquel Sánchez-Pérez, Luigi Ricciardi, Concetta Lotti

**Affiliations:** 1 Department of Soil, Plant and Food Science, University of Bari Aldo Moro, Via Amendola 165/A, Bari, 70126 Italy; 2 Institute of Biomedical Technologies, National Research Council (CNR), Via Amendola 122/D, Bari, 70126 Italy; 3 Department of the Sciences of Agriculture, Food and Environment, University of Foggia, Via Napoli 25, Foggia, 71100 Italy; 4 Council for Agricultural Research and Economics-Research Centre for Agriculture and Environment (CREA-AA), Bari, 70125 Italy; 5 Department of Agricultural Sciences, University of Naples Federico II, Portici, 80055 Italy; 6 Institute of Biosciences and Bioresources, National Research Council of Italy, Portici, 80055 Italy; 7 CEBAS-CSIC. Campus Universitario de Espinardo, 30100 Espinardo, Spain

**Keywords:** Plant breeding, Natural variation in plants, Agricultural genetics

## Abstract

Almond [*Prunus dulcis* Miller (D.A. Webb)] is the main tree nut species worldwide. Here, genotyping-by-sequencing (GBS) was applied to 149 almond cultivars from the ex situ collections of the Italian Council for Agricultural Research (CREA) and the Spanish National Research Council (CSIC), leading to the detection of 93,119 single-nucleotide polymorphisms (SNPs). The study of population structure outlined four distinct genetic groups and highlighted diversification between the Mediterranean and Californian gene pools. Data on SNP diversity and runs of homozygosity (ROHs) allowed the definition of kinship, inbreeding, and linkage disequilibrium (LD) decay in almond cultivated germplasm. Four-year phenotypic observations, gathered on 98 cultivars of the CREA collection, were used to perform a genome-wide association study (GWAS) and, for the first time in a crop species, homozygosity mapping (HM), resulting in the identification of genomic associations with nut, shell, and seed weight. Both GWAS and HM suggested that loci controlling nut and seed weight are mostly independent. Overall, this study provides insights on the almond cultivation history and delivers information of major interest for almond genetics and breeding. In a broader perspective, our results encourage the use of ROHs in crop science to estimate inbreeding, choose parental combinations minimizing the risk of inbreeding depression, and identify genomic footprints of selection for specific traits.

## Introduction

Almond (*Prunus dulcis* Miller (D.A. Webb), syn. *Prunus amygdalus* L., 2*n* = 2x = 16) is one of the oldest domesticated tree species, presumably originating in the first half of Holocene^1^. Starting from the Fertile Crescent, almond cultivation rapidly spread westwards and eastwards through human migration and commercial routes. At present, almond is the main tree nut species worldwide, with an estimated production of 2.2 million tonnes (FAOSTAT data 2017). Approximately 80% of the global almond cultivated area is concentrated in California and the Mediterranean region (FAOSTAT data 2017). Clonal cultivars are widely grown in developed countries, whereas seedling populations mainly occur in developing countries.

Information on the genetic structure of crop species is pivotal for the correct management of ex situ germplasm collections and the efficient implementation of breeding programs, which should be based on the hybridization of genetically diverse individuals^2^. In addition, geographic patterns in the genetic structure may reveal key events, including routes of dissemination, associated with crop cultivation histories^3,4^. So far, studies aiming at the characterization of the almond genetic structure used a few simple sequence repeat (SSR) markers^5–9^. Nowadays, high-throughput genotyping methods, such as genotyping-by-sequencing (GBS), are routinely applied in agrigenomics research for the fine-scale characterization of genetic structure with thousands of single-nucleotide polymorphism (SNP) markers^10–12^.

Inbreeding depression, i.e. the reduced fitness of offspring of related individuals^13^, has been widely documented in human, animal, and plant populations. In almond, inbreeding depression leads to declined vegetative vigor and dramatic reduction of flower and fruit set^14–16^. Therefore, information on kin relationships among cultivars, which can be reconstructed from pedigrees or inferred a posteriori from genotypic data^17,18^, is of great value for almond breeders to minimize the risk of inbreeding depression. In addition, it would be interesting to investigate whether some of the almond cultivars currently grown on a large scale are inbred, and thus may display deleterious phenotypes due to inbreeding depression.

The level of inbreeding of an individual (F) depends on the extent of homologous chromosome segments displaying identity by descent (IBD), i.e., deriving from the same recent common ancestor^19^. Therefore, F can be estimated by the pedigree inbreeding coefficient (F_P_), which expresses IBD probabilities based on pedigree data. However, the use of F_P_ has two major limitations, i.e., the strong assumption that the pedigree founders are unrelated, and the difficulty to retrieve pedigree information^20^. Another estimator of F (here named F_PLINK_, as it can be calculated by the widely used PLINK bioinformatics toolset) is based on the positive correlation occurring between IBD and the ratio between homozygosity observed and expected at Hardy–Weinberg equilibrium^17^.

To date, high-throughput genotyping enables the assessment of inbreeding through the direct detection of IBD segments. These appear as long chromosomal stretches of homozygous marker loci, referred to as runs of homozygosity (ROHs). ROHs were proven to be more accurate than F_P_ when genotyping with thousands of SNP markers^19^ and are therefore commonly used to estimate inbreeding in human and cattle population studies^21,22^. In contrast, only a few works used ROHs to estimate inbreeding in plants^23^.

Seed and nut weight are economically important traits associated with the almond cultivation and processing industry. Specifically, seed weight is a major determinant for the almond final market utilization, whereas nut weight, which is given by the sum of the seed and shell weight, is important to set up appropriate harvesting, dehulling, transportation, and storing strategies^24^. Previous studies indicated that seed and nut weight are highly heritable and weakly correlated with the production density^25–27^. In addition, QTLs significantly associated with almond seed and nut weight were mapped on chromosomes 1, 2, and 7^24^.

The recent publication of the almond genome sequence^28,29^ provides the opportunity to carry out genome-wide association studies (GWASs), identifying associations between phenotypes and markers with the known chromosomal locations. Information on the average linkage disequilibrium (LD) decay in the organism on which a GWAS is performed is of main importance, as this parameter influences, for a given number of markers, the chance to reveal significant associations^12,30^. In addition, rapid LD decay increases the possibility that GWAS experiments lead to the identification of marker loci residing within, or in the proximity of, genes causally related to the phenotype.

Besides GWAS, homozygosity mapping (HM) is another approach enabling the disclosure of genotype–phenotype relationships, which consists in testing the association between traits of interest and ROHs^31^. Compared to GWAS, HM has the major advantage of using a lower number of covariates, thus reducing type II error associated with multiple correction tests. Practically, this means that HM may reveal genomic associations that escape GWAS detection. In addition, IBD identified by HM can highlight genomic regions associated with inbreeding depression or selective pressure^32^. Indeed, in medical genomics, HM is performed to map homozygosity derived from consanguinity and associated with recessive diseases^33^, whereas, in animal science, it is carried out to highlight genomic regions resulting from human selection for specific traits^32,34^. To the best of our knowledge, HM has been never applied in plant research.

Here, we aimed to study genetic structure, kinship, inbreeding, and average LD decay in almond cultivated germplasm, through GBS-based identification of SNPs and ROHs. In addition, we addressed the detection of genomic regions associated with nut, shell, and seed weight, based on HM and GWAS.

## Results

### GBS results and quality control

Sequencing of a GBS library based on 149 almond cultivars (Supplementary Table S1) generated about 2.5 million reads/sample. Mean read depth in individual cultivars is reported in Supplementary Fig. S1. On average, SNP calling was supported by 47.08 reads per locus.

The SNP filtering procedure generated 93,119 markers, resulting in an average density of one SNP/2.18 Kb. The almond chromosomes 1–8 contained 18,357, 12,244, 10,733, 10,559, 8818, 12,680, 9679, and 10,049 SNPs, respectively. The cultivars Del Cid, Peraleja, and Lauranne were excluded from downstream analyses, as they displayed low genotypic call rates.

Based on pairwise identity by state (IBS) distance among biological replicates of the same cultivar, 11 clonal groups (CG1–11) were identified (Supplementary Table S2). For each clonal group, the cultivar associated with the highest SNP call rate was selected to represent the group, leading to a panel of 131 genetically distinct cultivars used for further analyses.

### Population structure

**Fig. 1:**
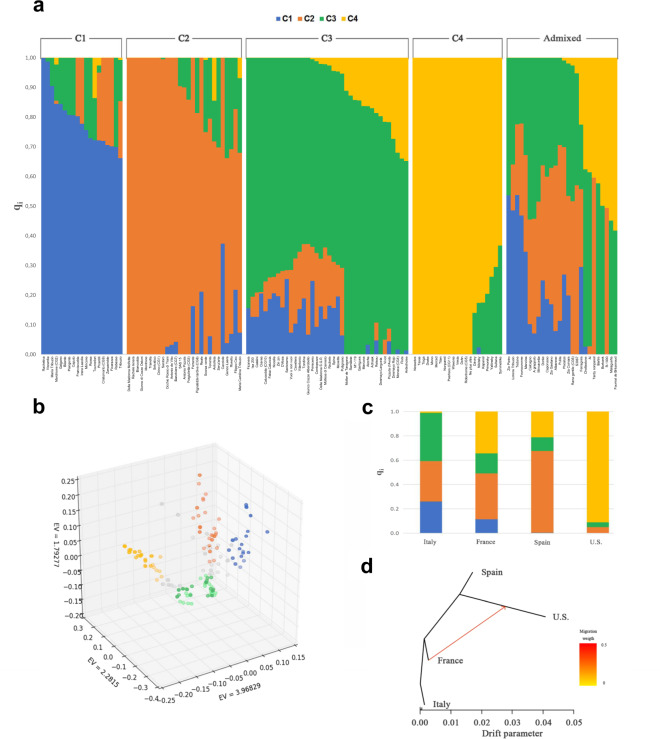
Genetic structure of almond cultivated germplasm. **a** Results of ADMIXTURE analysis for *K* = 4. Each cultivar is represented by a vertical bar. The length and the color of segments in each bar represent the proportion of the genome (qi) contributed by each of four ancestral populations (C1–C4). Cultivars assigned to one of the populations have a membership coefficient (qi) for that population >0.6. The remaining cultivars are classified as admixed. **b** Scatter plot for genetic variation explained by the top three principal components. Cultivars assigned to C1–C4 are colored in accordance with panel **a**. Admixed individuals are colored in gray. **c** Stacked bar plots indicating the ancestry of the Italian, French, Spanish, and U. S. almond populations, based on qi coefficients resulting from ADMIXTURE analysis. **d** Treemix output for the Italian, French, Spanish, and U. S. almond populations. Branch lengths represent the evolutionary change as resulting from the drift parameter. The migration weight describes the percentage of ancestry derived from the source population. The scale bar represents the average standard error (se) of the values of the covariance matrix

Analysis with the parametric clustering method implemented by the software ADMIXTURE^35^ indicated that a model with four ancestral populations (C1–C4) was the most suitable to describe genetic structure (Supplementary Fig. S2). Based on their membership coefficient (q_i_), 105 cultivars were assigned to one of the ancestral populations, whereas 26 cultivars were assigned to the admixed group. C1 and C2, composed by 19 and 27 cultivars, respectively, mainly include Italian germplasm; C3 encompasses 38 cultivars originating from several Mediterranean countries; finally, C4 is formed by 21 cultivars, all from U.S and Ukrainian origin, except for the French cultivar Sultana and the Greek cultivar Symmetrike (Fig. 1a). Support to the ADMIXTURE results was provided by a nonparametric study of the genetic structure by principal component analysis (PCA), as the first three principal components clearly differentiated cultivars assigned to different ancestral populations (Fig. 1b).

The French population displayed significant contributions from all the four ancestral populations C1–C4. Conversely, the Italian and Spanish populations displayed minimal contribution (<1%) from C4 and C1, respectively, and the U.S. population could be mostly referable to C4 (Fig. 1c). The modeling approach implemented in TreeMix^36^ highlighted the largest genetic distance between Italian and U.S. germplasm. Strong support was found for a model with one migration event between French and U. S. germplasm (Fig. 1d), which, compared with a model with no migration, increased the percentage of explained allele frequency covariance among populations from 0.97 to 1.

### Kin relationships among cultivars

**Fig. 2:**
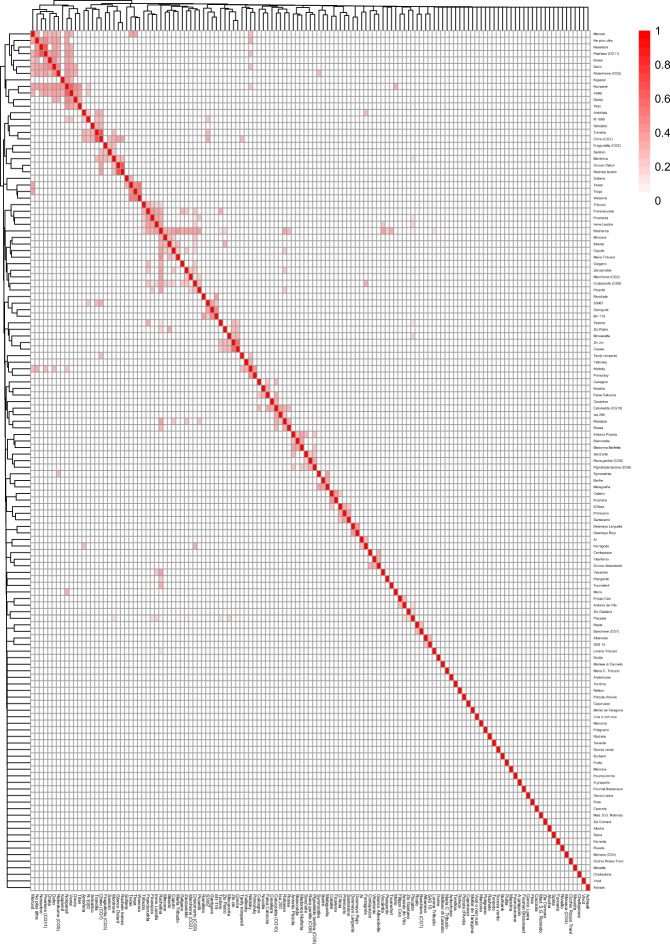
Heatmap produced on estimated pairwise IBD values among almond cultivars. Cladograms were produced with the unweighted pair group method with arithmetic mean (UPGMA) hierarchical clustering algorithm

Kin relationships were predicted for known parent/offspring pairs present in the cultivar collection (“Cristomorto” (CG9)/”Ferragnès”, “Aï’/‘Ferragnès”, “Ferragnès”/”Antoñeta”, “Chino” (CG1)/“Antoñeta”, “Texas”/”Merced”, “Nonpareil”/”Merced”, “Nonpareil”/”Davey“, and “Nonpareil”/”Kapareil”) (Supplementary Table S1), with PI_HAT values ranging from 0.26 to 0.45. Several family clusters were identified, of which the largest included the U.S. cultivars Davey, Dhen, Drake, Kapareil, Merced, Ne Plus Ultra, Nonpareil, Peerlees (CG11), Ridenhome (CG5), Titan, and Vesta (Fig. 2). The Italian cultivar Rachelina displayed the highest number of kin relationships (22), with PI_HAT values ranging from 0.06 to 0.41 (Fig. 2 and Supplementary Table S3). The French cultivars Sultana and R1000, the Greek cultivar Symmetrike, and the Italian cultivar Chino (CG1) were the only Mediterranean cultivars displaying kinship with U.S. germplasm.

### Estimation of individual inbreeding

**Fig. 3:**
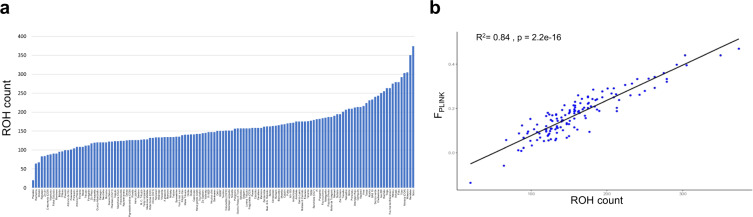
Study of inbreeding in almond cultivated germplasm. **a** Ranking of cultivars based on the count of runs of homozygosity (ROHs). **b** Linear regression model between ROH count and the PLINK inbreeding coefficient (F_PLINK_)

In total, 21,019 ROHs were detected in the panel of cultivars, leading to an average of 160.45 ROHs/cultivar. In Supplementary Fig. S3, the distribution of the percentage of missing data per ROH is reported. Weak correlation (adjusted *R*^2^ = 0.16) was found between mean read depth per cultivar and ROH count per cultivar (Supplementary Fig. S4). Ranking according to ROH count indicated a high level of inbreeding in several U.S. cultivars, with Mono displaying the highest ROH count (374) (Fig. 3a). The lowest ROH count (20) was observed in the Italian cultivar Piscalze. A similar ranking was obtained when sorting cultivars according to the cumulative ROH length, with the U.S. cultivar Wawona and the Italian cultivar Piscalze showing the highest (82.9 Mb) and the lowest (2.9 Mb) values, respectively (Supplementary Fig. S5). A strong correlation (adjusted *R*^2^ = 0.84) was found between ROH count and F_PLINK_ (Fig. 3b).

### Almond average LD decay

**Fig. 4:**
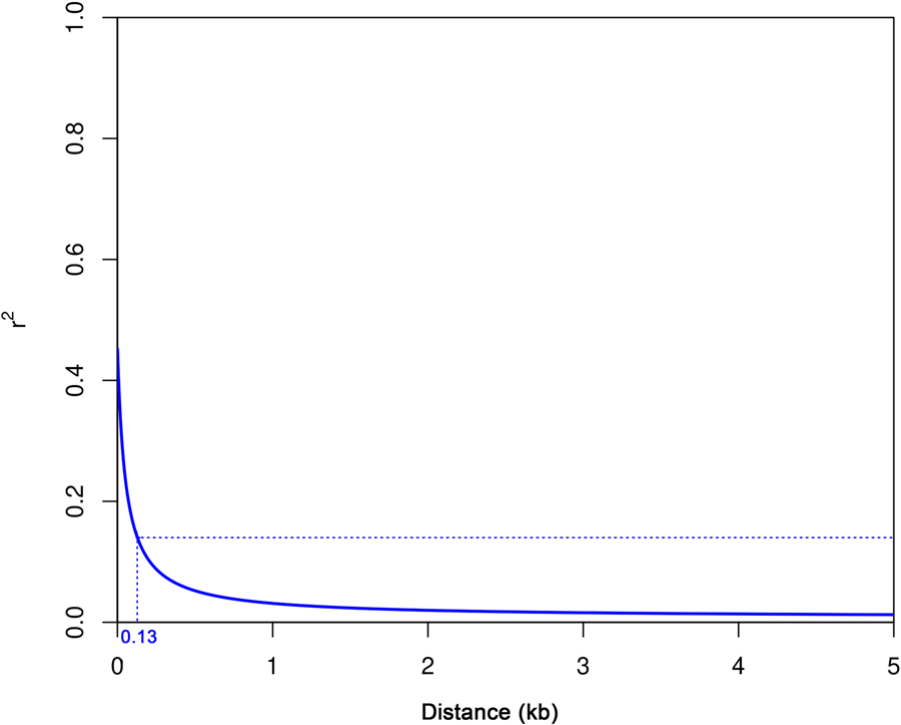
Pattern of LD decay. On average, *R*^2^ = 0.141, assumed as the lower threshold to declare LD between two loci, is reached after 130 bp

Correlation of the allelic state at pairs of different loci, expressed through the pairwise squared correlation coefficient *R*^2^, was equal to 0.141 on average. This value assumed as the lower threshold to declare LD between two loci was reached on average after 130 bp (Fig. 4). Mean pairwise *R*^2^ values were quite similar for marker loci within individual chromosomes, ranging from 0.132 (chromosome 6) to 0.147 (chromosome 2) (Supplementary Table S4).

### Homozygosity mapping and genome-wide association study

The distribution of best linear unbiased predictors (BLUPs) calculated for nut, shell, and seed weight is reported in Supplementary Fig. S6. The null hypothesis of normal distribution could be accepted for all the traits (Kolmogorov–Smirnov test *P* value > 0.05). A high correlation was found between nut and shell weight (adjusted *R*^2^ = 0.96), whereas a weak correlation was found between nut and seed weight (adjusted *R*^2^ = 0.1). No significant correlation was found between shell and seed weight (Supplementary Fig. S7).

**Fig. 5:**
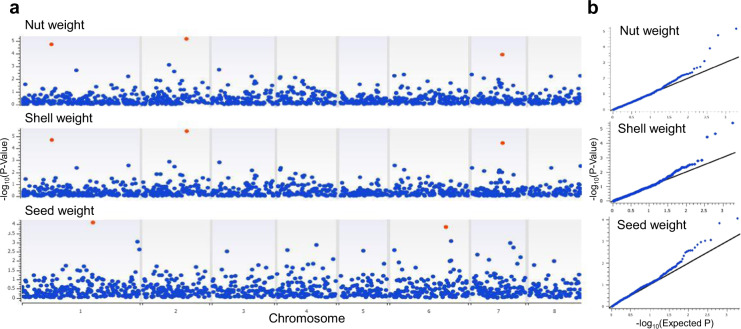
Homozygosity mapping (HM) for nut, shell, and seed weight. **a** Genomic distribution of clusters of runs of homozygosity (ROHs), whose loci occur in ROHs identified in at least ten cultivars. Red dots indicate association for FDR-corrected *P* value < 0.1. **b** Q–Q plots relative to the regression models

HM suggested an association between nut weight and three clusters of ROHs located on the almond chromosomes 1, 2, and 7 (Fig. 5 and Supplementary Table S5), with the cluster ROH_2_16414730 displaying the highest indication of significance (−log_10_ (*P* value) = 5.17; false discovery rate (FDR) *P* value = 6 × 10^−3^). In accordance with the correlation pattern found for phenotypic data, the same clusters of ROHs associated with nut weight were also associated with shell weight (Fig. 5 and Supplementary Table S5), but not with seed weight. Indication of association with seed weight was found for two ROHs on chromosomes 1 and 6 (Fig. 5 and Supplementary Table S5). Notably, cultivars contributing to the clusters of ROHs identified by HM displayed significantly higher nut or seed weight (Table 1), indicating that such homozygous regions might represent genomic footprints of selection for larger nuts and seeds.

**Table 1:** Comparison of nut and seed weight BLUP means calculated for cultivars contributing (+) or not contributing (−) to clusters of ROHs identified by HM analysis

		Mean ± se ROH ( + )	Mean ± se ROH (-)	*P* value
Nut weight	ROH_S1_11260515	1.36 ± 1.12	−0.12 ± 0.10	6.37E-04
	ROH_S2_16414730	1.43 ± 0.46	−0.22 ± 0.10	1.29E-06
	ROH_S7_11701812	1.62 ± 0.96	−0.11 ± 0.11	4.86E-04
Seed weight	ROH_S1_26367663	0.14 ± 0.06	−0.03 ± 0.02	8.80E-04
	ROH_S6_20767156	0.36 ± 0.13	−0.02 ± 0.02	2.85E-05

For each comparison, *P* values associated with two-tailed *t* test are reported.

**Fig. 6:**
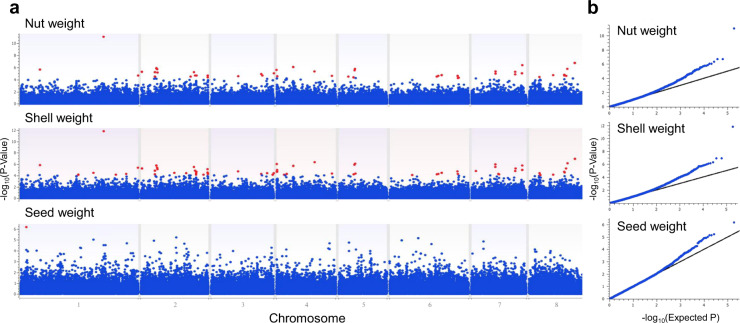
Genome-wide association study (GWAS) for nut, shell, and seed weight. **a** Manhattan plots. Red dots indicate association for FDR-corrected *P* value < 0.1. **b** Q–Q plots relative to the regression models

In total, 57 GWAS signals were identified for nut weight (Fig. 6 and Supplementary Table S6). By far, the highest indication of association (-log_10_ (*P* value) = 11.05; FDR *P* value = 8.22 × 10^−7^) was found for the marker S1_30936643, residing in the putative promoter sequence (218 bp upstream the start codon) of the aspartyl protease gene *Prudu_003450_v1.0*. In accordance with the results of HM, SNP loci associated with nut weight were in most cases also associated with shell weight (Fig. 6 and Supplementary Table S6). A single GWAS signal (−log_10_ (*P* value) = 6.19; FDR *P* value = 0.06) was detected for seed weight, for a marker (S1_2496687) located within the gene *Prudu_000307_v1.0*, encoding a putative pathogenesis-related thaumatin superfamily protein (Fig. 6 and Supplementary Table S6).

## Discussion

In this study, we report the characterization of genome-wide diversity and homozygosity in almond, which were used to provide information of major interest for fundamental research and breeding purposes.

Calculation of pairwise IBS distances resulted in the identification of 11 clonal groups (Supplementary Table S2), thus providing valuable data for the rationalization of germplasm collections. The composition of CG1 is in accordance with previous scientific literature, indicating synonymy between the cultivars Tuono and Troito^37^. We cannot exclude that some of the cultivars included in the same CG may differ for a few clonal mutations. Concerning CG1, it was previously reported that the cultivar Supernova was derived from “Tuono” by experimental mutagenesis^38^.

Analysis of almond genetic structure assigned cultivars to four ancestral populations (Fig. 1a), although caution should be taken in such interpretation of ADMIXTURE clustering results^39^_._ Cultivars classified as admixed, corresponding to ~20% of the total, might reflect hybridization between different ancestral populations. It is generally thought that almond was introduced to Italy by ancient Greeks and Phoenicians^37^, and from there spread to France and Spain, possibly through Ancient Romans expanding in the Mediterranean area. However, the Italian almond population almost completely lacked the C4 ancestry, which was significant for the Spanish and French gene pools. This evidence can be explained by further historical introductions of almond germplasm to Spain and France, possibly from North Africa in relation to the Arabic domination of the Iberic Peninsula and the colonial period^37^.

In accordance with previous investigations based on SSR markers^6–8^, the study of population structure also indicated genetic differentiation between Mediterranean and U.S. cultivars, with these last mostly referable to the ancestral cluster C4 (Fig. 1). This result most likely reflects the founder effect associated with the recent introduction of almond to the New World. Importantly, migration modeling using the TreeMix algorithm (Fig. 1d) indicated that French germplasm played an important role as a parental source for Californian almond breeding, in accordance with previous literature^40–42^.

Estimation of pairwise IBD through the PI_HAT parameter correctly indicated kinship for known parent/offspring cultivar pairs occurring in the almond collection genotyped in this study, as these were associated with values ranging from 0.26 to 0.45. The discrepancy with the theoretical PI_HAT value of 0.5 (i.e., 50% of the alleles originating from the same ancestral chromosomes) might be due to the violation of the assumption of random mating used for PI_HAT estimation^17^ and under-calling of heterozygous loci associated with the GBS method^43^. Besides confirming known kinship, IBD analysis unveiled several familial relationships that were not reported in the literature (Fig. 2 and Supplementary Table S3). This result, besides contributing to uncover the pedigree of almond cultivated germplasm, provides useful information to avoid hybridization of related individuals in breeding programs, thus minimizing the risk of inbreeding depression.

A large family group was composed by several U.S. cultivars, including “Nonpareil”. This is consistent with the recurrent use of “Nonpareil”, considered as standard for superior tree and nut characteristics, in U.S. breeding programs^14,44^. Several cultivars displayed kinship with CG1 and CG9, containing the Italian cultivars Tuono and Cristomorto, in accordance with the extensive use of these two cultivars in breeding as a source of self-compatibility. Surprisingly, the Italian cultivar Rachelina, which is not reported in main pedigree records, displayed the highest number of kin relationships (22), not only with Italian germplasm, but also with the French cultivars Rabasse and Tournefort, and the Ukrainian cultivar Picantili. The identification of kinship between “Sultana” and “Texas” further indicates the role of French introductions as founders of U.S. breeding programs. Remarkably, “Sultana” was previously indicated as one of the few commercial cultivars introduced to California from the Languedoc area of Southern France from 1850 to 1900, representing the basis of the U.S. almond industry^37,41,45^. Finally, the kinship between Ukrainian cultivars (“Crimsky”, “Nikitsky”, “Nessebre”, “Picantili”) and cultivars from Italy and U.S. is consistent with the use of foreign germplasm in breeding programs held at the Ukrainian Nikita Botanical Garden^46,47^.

Similarly to the work by Wu et al.^23^, which focused on cultivated *Citrus* species, we searched for ROHs to estimate the level of inbreeding in individual cultivars (Fig. 3a and Supplementary Fig. S5). A high correlation was found between ROH count per individual and F_PLINK_ inbreeding coefficient (Fig. 3b). However, we stress that, differently from ROHs, F_PLINK_ is an indirect estimator of F, based on increased homozygosity associated with IBD. Several U. S. cultivars were characterized by high ROH count and length, indicating a high level of inbreeding. This is in accordance with our finding that a high level of IBD occurs within U.S. germplasm. In contrast, Lansari et al.^14^, based on the F_P_ coefficient, concluded that most U.S. cultivars are non-inbred, possibly due to incomplete pedigree information.

It is known that one of the major technical drawbacks of GBS is uneven read depth among samples^43^. To evaluate whether this generated a severe bias in heterozygous loci, and thus ROH, calling, we performed a regression analysis between mean read depth per cultivar and ROH count per cultivar. We found a weak correlation between the two variables, although two cultivars, “Mono” and “Ramillete”, associated with extremely low mean read depth, also displayed the highest ROH count (Supplementary Fig. S4). This indicates that: (1) with a few exceptions, our GBS approach was successful in quantifying the level of inbreeding through ROH identification; (2) when available, SNP array platforms, allowing accurate heterozygosity call, should be preferred over GBS to identify ROHs. Missing data did not have a major impact on ROH call, as most ROHs contained a low percentage of missingness (Supplementary Fig. S3).

Homozygosity mapping^31^, a strategy successfully used in animal science to associate ROHs with traits under anthropic selection^34,48^, was herein applied for the first time to a crop species. Our results defined IBD segments which could have arisen from selection for larger nuts and seeds. In addition, our data suggest that selection for larger nuts, while increasing the weight of the fruit endocarp (the almond shell), did not have a substantial effect on the almond edible part, i.e., the seed (Fig. 5). ROH_ 2_16414730, displaying the highest evidence of association with nut and shell weight, includes two members of the PLAC8 protein family, previously associated with fruit size in tomato, maize, and rice^49^ (Supplementary Table S5). Concerning seed weight, an interesting candidate for future functional studies is a Cyclin D3 gene located within ROH_S6_20767156, as it was shown that D-type cyclins play a major role in seed development^50^.

In accordance with the results of HM, GWAS suggested that loci controlling nut weight and seed weight are mostly independent and that there is parallel control of nut and shell weight by several genomic loci (Fig. 6). Many of the GWAS peaks identified for these two traits were located within genes encoding transcription factors or response factors to the phytohormones abscisic acid, auxin and ethylene (Supplementary Table S6). These protein categories are renowned to be major players in fruit growth and development^51^, therefore they are obvious candidates to have a similar physiological role in almond. The highest significance level for nut and shell weight was found for a SNP variant located upstream of a putative aspartyl protease gene (Supplementary Table S6). Remarkably, the recent proteomic study by Rodriguez et al.^52^ indicated that the development of the peach endocarp (corresponding to the almond shell), is accompanied by an outstanding variation of protein degradation enzymes, including aspartyl proteases. It is thought that amino acids derived from the degradation of proteins stored in the early immature fruit act as substrates for the phenylpropanoid and lignin pathways activated during endocarp hardening^53^.

Concerning seed weight, the association was found with a SNP residing in a gene putatively encoding a member of the thaumatin-like protein (TLP) superfamily (Supplementary Table S6). Although some of the TLP proteins have been related to biotic stresses, the role of most members of the TLP superfamily remains unknown^54^, thus it cannot be excluded they might also have a role in determining seed growth. With this respect, we highlight that some TLP proteins, referred to as permeatins, accumulate in high concentration in seeds of cereals^54,55^.

No overlap was found between genomic regions identified by GWAS and HM. It should be pointed out that GWAS and HM search for different kind of genomic associations, in the first case with a specific marker allele, and in the second with one or more combinations of alleles at the homozygous state. In addition, different results from the two approaches may arise from the different number of covariates used for association tests. We could not assess whether signals on the same chromosome identified by our study and the one of Fernandez i Marti et al.^24^ are overlapping in the same genomic region, as the latter refers to a QTL linkage map obtained by a bi-parental population, rather than the almond genome sequence.

We found that almond displays one of the fastest LD decay ever characterized in a crop species, with *R*^2^ dropping to the threshold value after 130 bp on average (Fig. 4). This might reflect self-incompatibility displayed by most almond cultivars, which favors haplotype block-breaking through recombination. From a genetic perspective, rapid LD decline in almond reinforces the possibility that SNPs identified by this or future GWAS experiments are located within or in close association with genes determining phenotypic variation.

## Materials and methods

### Plant material

Plant material selected for this study includes 149 cultivars, of which 132 from the ex situ collection of CREA-AA (Italian Council for Agricultural Research and Analysis of Agricultural Economics—Section Agriculture and Environment), Bari, Italy, and 17 from the *ex situ* collection of CEBAS-CSIC (Spanish National Research Council—Center for Edaphology and Applied Biology of the Segura River), Murcia, Spain (Supplementary Table S1). Pedigree information, available for the cultivars Antoñeta, Davey, Ferragnés, Kapareil, and Merced, is reported in Supplementary Table S1.

### GBS assay and quality control

Leaf tissue samples were collected from three biological replicates of the cultivar R1000, two biological replicates of the cultivars Ardechoise, Ferragnès, Filippo Ceo, Marcona, and Desmayo Largueta, and one individual of the remaining cultivars. DNA was extracted using the DNeasy Plant Mini Kit (Qiagen) and assayed for quality and concentration using agarose gel (0.8%) electrophoresis and the Qubit 3.0 fluorometer (Life Technologies). A GBS library was prepared as reported by Elshire et al.^10^, using the restriction enzyme *Ape*KI (The Elshire Group Ltd.). Paired-end sequencing was performed using the HiSeq2500 device (Illumina), including an empty negative control well. The TASSEL-GBS pipeline^43^ and the almond reference genome^28^ were used for SNP calling and mapping.

Quality control was carried out using TASSEL v.5^56^. Specifically, marker quality control was performed by filtering for biallelic SNP loci with minor allele frequency >0.05 and call rate >0.7. As for genotype quality control, cultivars associated with an overall SNP call rate >0.6 were selected. In addition, mean and sd of the pairwise IBS distance^17^ between biological replicates were used to set up an IBS minimal threshold (mean−3 × sd) to declare clonal groups and select, within each group, the cultivar with the highest SNP call rate.

### Analysis of population structure

Genetic structure was studied using SNPs in approximate linkage equilibrium, which were obtained using the LD pruning algorithm in PLINK v.1.90p^17^. This calculates pairwise *R*^2^ for all marker pairs in sliding windows with a size of 50 markers and an increment of 5 markers and removes the first marker of pairs, in which *R*^2^ < 0.5.

Analysis with the ADMIXTURE parametric model^35^ was performed with a number of ancestral populations (*K*) ranging from 1 to 15. One thousand bootstrap replicates were run to estimate parameter standard errors. The most suitable number of *K* was selected in correspondence with the lowest cross‐validation (CV) error. Cultivars were assigned to one specific ancestral population when the membership coefficient q_i_ for that cluster was >0.6. If not, they were considered admixed.

PCA on SNP data was performed using SVS v.8.8.3 (Golden Helix Inc.), and a three-dimensional plot was obtained using the top three components identified with default parameters of the additive model.

TreeMix (v1.12)^36^ was used to infer splits and mixtures among Italian, French, Spanish, and U.S. germplasm, testing a model with no migration, and models with all the three possible migration events among the four populations. The “get_f()” R function was used to obtain the variance explained by each model.

### Inference of kinship

In order to infer kinship among cultivars, pairwise genotype probabilities (*P*) of sharing 0, 1, or 2 IBD alleles were calculated at each locus, given IBS distances and allele frequencies, using the method-of-moments algorithm implemented in PLINK v.1.90p^17^. The matrix obtained with the PI_HAT parameter, given by P(IBD = 2) + 0.5 × P (IBD = 1) and providing an estimate of the proportion of IBD alleles, was used to draw a clustered heatmap, using the pheatmap v.1.0.12. R package^57^.

### ROH detection and quantification of individual inbreeding

ROH detection analysis was carried out with a subset of SNPs, selected for having MAF > 0.15. The algorithm implemented in SVS v.8.8.3 (Golden Helix Inc.) was used to identify completely homozygous genomic stretches on chromosomes 1–8 with at least 15 SNP loci and with a minimal length of 100 Kb. As a measure of inbreeding, the ROH count and the ROH total length were computed for each individual. The ggplot2 R package^58^ was used to visualize the distribution of the percentage of missing data per ROH and to perform a regression analysis between mean read depth per cultivar and ROH count per cultivar.

Individual inbreeding was also estimated using the F_PLINK_ inbreeding coefficient, which was computed using the LD-pruned marker dataset as input. Regression analysis between ROH count, or ROH total length, and F_PLINK_ coefficient, was performed using the ggplot2 R package^58^.

### Estimation of LD decay

PLINK v.1.90p^17^ was used to calculate pairwise linkage disequilibrium (LD) between SNPs^59^, expressed as the squared correlation coefficient *R*^2^. The *R*^2^ values were plotted against the marker physical distance, and the Hill and Weir formula was used to describe the decay of r^2^^60^. The mean pairwise *R*^2^ for markers within and between chromosomes was used to define a lower threshold value for LD. Mean pairwise *R*^2^ was also calculated for markers on individual chromosomes.

### Phenotypic data collection and analysis

Phenotyping was carried out in 2006, 2007, 2009, and 2012, on 98 cultivars of the CREA-AA collection, grown at the experimental farm “La Piantata” (Bari, Italy) (41°02’29.9”N; 16°46’01.4”E, 126 m a.s.l.) (Supplementary Table S1). The orchard was established in 1968 according to a completely randomized design with three clonal replicates for each cultivar. Plants were grown according to traditional practices in Southern Italy, without irrigation, and trained as classic vase. Nut and seed weight were determined for each cultivar as the average of its three clonal replicates. In turn, data from each clone were determined as the average of thirty fruits. Data on the shell weight were obtained by the difference between nut and seed weight.

BLUPs of phenotypic traits collected over 4 years were calculated using the Lme4 R package^61^ and the following model: y = lmer (Trait ~ (1 | Genotype) + (1 | Year)). Normal distribution of BLUP data was verified using the Kolmogorov–Smirnov test implemented in the stats R package. Linear models to study correlation between BLUPs for nut, shell, and seed weight were generated using the ggplot2 R package^58^.

### Homozygosity mapping

HM was carried out using options available in SVS v.8.8.3 (Golden Helix Inc.). Clusters of ROHs, defined as genomic regions of at least 100 Kb whose loci occur in ROHs of at least ten cultivars characterized at the phenotypic level, were identified. Repeated binary spectral clustering^62^ was used to trim boundaries of clusters of ROHs, in order to define homozygous regions highly overlapping among cultivars. Finally, a linear regression model was fit between clusters of ROHs and BLUPs, using the top five principal components as covariates to correct for population structure. The FDR correction was used to account for multiple testing and suggest an association for *P* < 0.1. BLUP means of cultivars either contributing or not contributing to clusters of ROHs associated with phenotypic traits were computed and compared using a two-tail Student’s *t* test. Genes included in clusters of ROHs identified by HM were retrieved by the *Prunus dulcis* (cv. Lauranne) v1.0 genome annotation available at the genomic database of Rosaceae^28,63^.

### Genome-wide association study

The EMMAX linear mixed model^64^ was used for GWAS, using BLUPs as phenotypic values and the IBS matrix as a covariance matrix of random effects. The FDR correction was used to suggest the association for *P* < 0.1. Genes containing or flanking SNPs associated with phenotypic traits were retrieved by the *Prunus dulcis* (cv. Lauranne) v1.0 genome annotation available at the genomic database of Rosaceae^28,63^.

## Supplementary information

Supplementary Figures

Supplementary Tables

## Data Availability

The unfiltered variant call format (VCF) file relative to the GBS experiment is publicly available at the Figshare repository (doi: 10.6084/m9.figshare.12205652).
