# Supplementary material for: Almond diversity and homozygosity define structure, kinship, inbreeding, and linkage disequilibrium in cultivated germplasm, and reveal genomic associations with nut and seed weight
Source: Hortic Res. 2021 Jan 10;8:15. doi: 10.1038/s41438-020-00447-1 (PMC7797004; doi:10.1038/s41438-020-00447-1)
Supplement: Supplementary file 1 — Supplementary Figures [file 41438_2020_447_MOESM1_ESM.docx]

## Supporting Information Figures

**Article title**: Diversity and homozygosity of almond define structure, kinship, inbreeding, and linkage disequilibrium in cultivated germplasm and reveal genomic associations with nut and seed weight

**Authors**: Stefano Pavan, Chiara Delvento, Rosa Mazzeo, Francesca Ricciardi, Pasquale Losciale, Liliana Gaeta, Nunzio D’Agostino, Francesca Taranto, Raquel Sánchez-Pérez, Luigi Ricciardi, Concetta Lotti

**Fig. S1** Mean read depth obtained from GBS library sequencing.

**Fig. S2** ADMIXTURE cross-validation (CV) error estimates for hypothetical clusters (K) ranging from 1 to 15.**
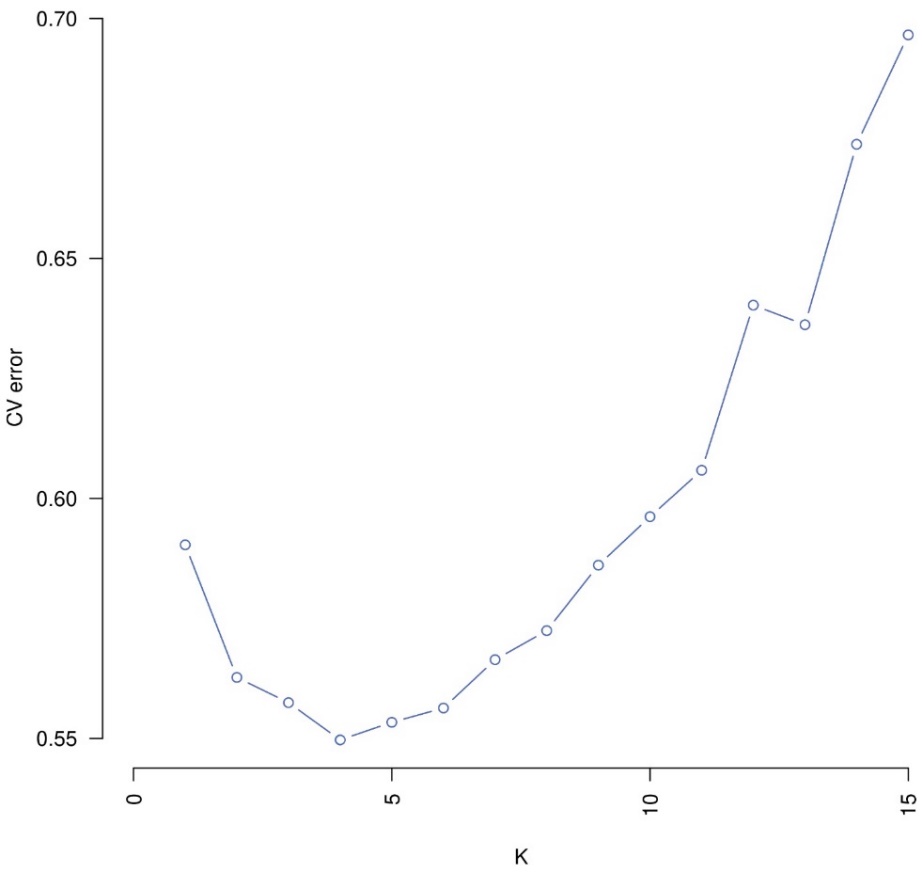
**

**Fig. S3** Frequency distribution of the percentage of missing SNPs per ROH.


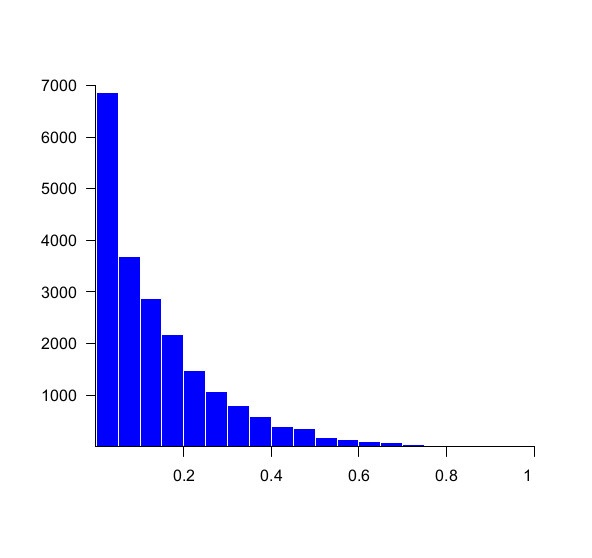


ROH count

**Fig. S4** Linear regression analysis between mean genomic read depth per cultivar and ROH count per cultivar.


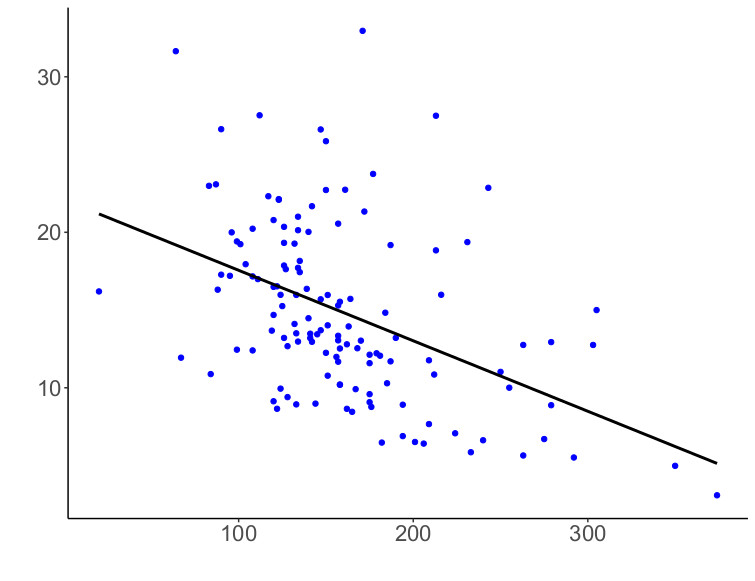


Mean genomic read depth

ROH count

R^2^ = 0.16, p= 9.6e-7

**Fig. S5** Ranking of cultivars based on cumulative ROH length.

**Fig. S6** Frequency distribution of BLUPs for nut (a), shell (b) and seed (c) weight among cultivars.

**
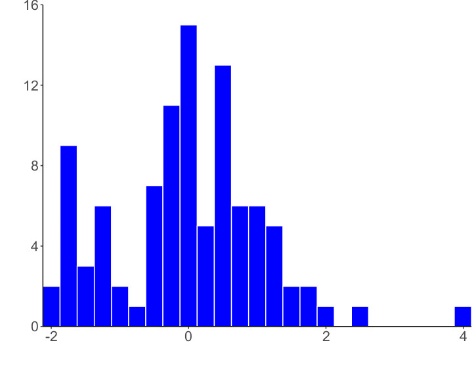

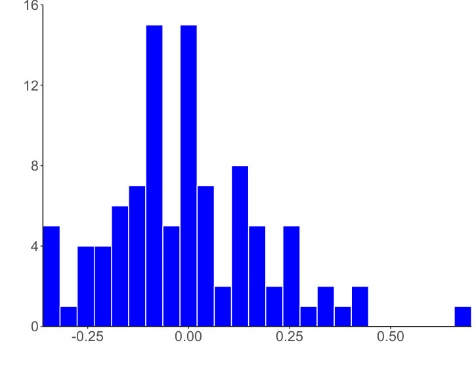

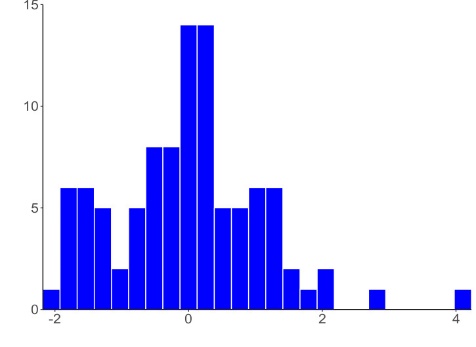
**

**a**

**b**

**c**

**Fig. S7** Linear regression analysis between nut weight and seed weight (a), nut weight and shell weight (b), shell weight and seed weight (c)**.**


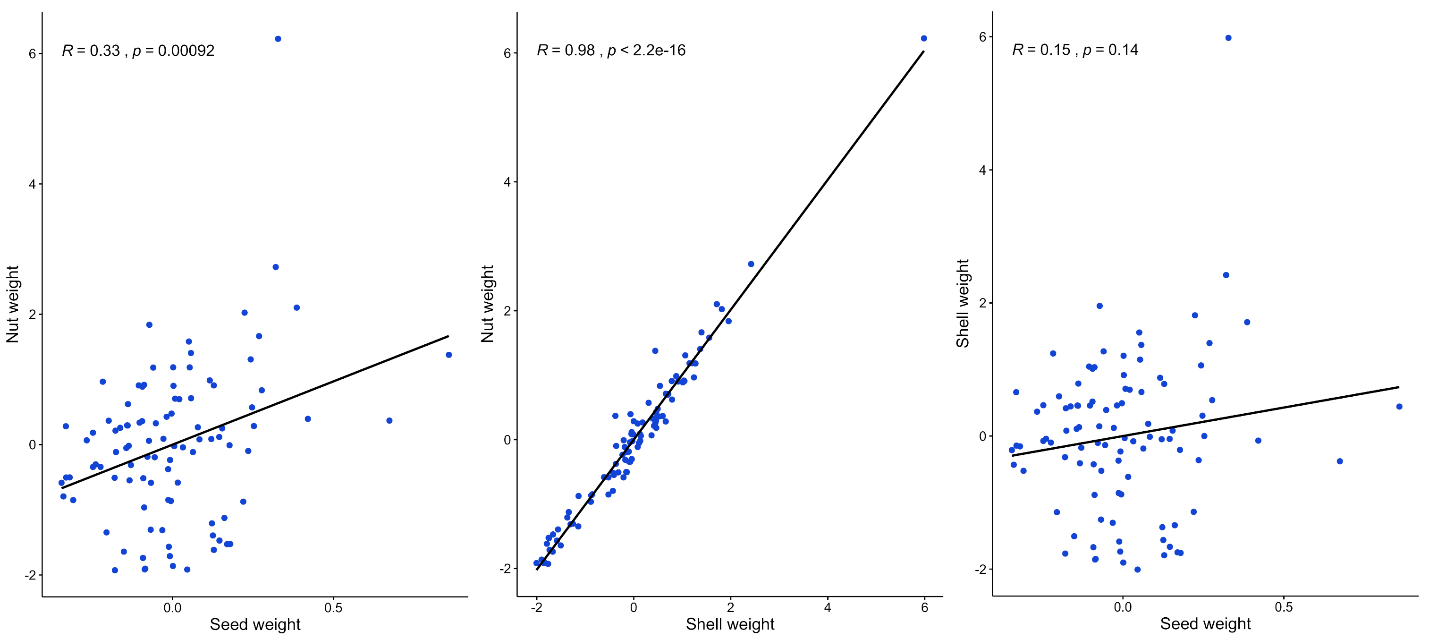

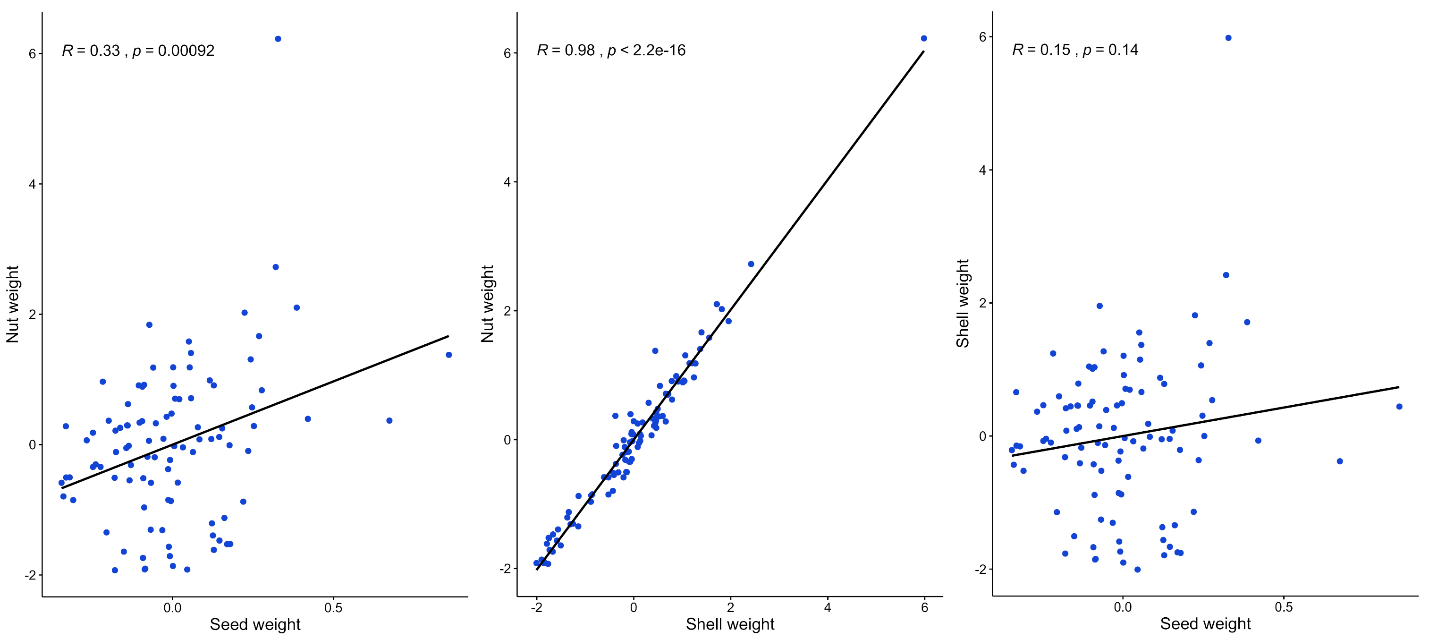


R^2^= 0.1, p = 0.00092


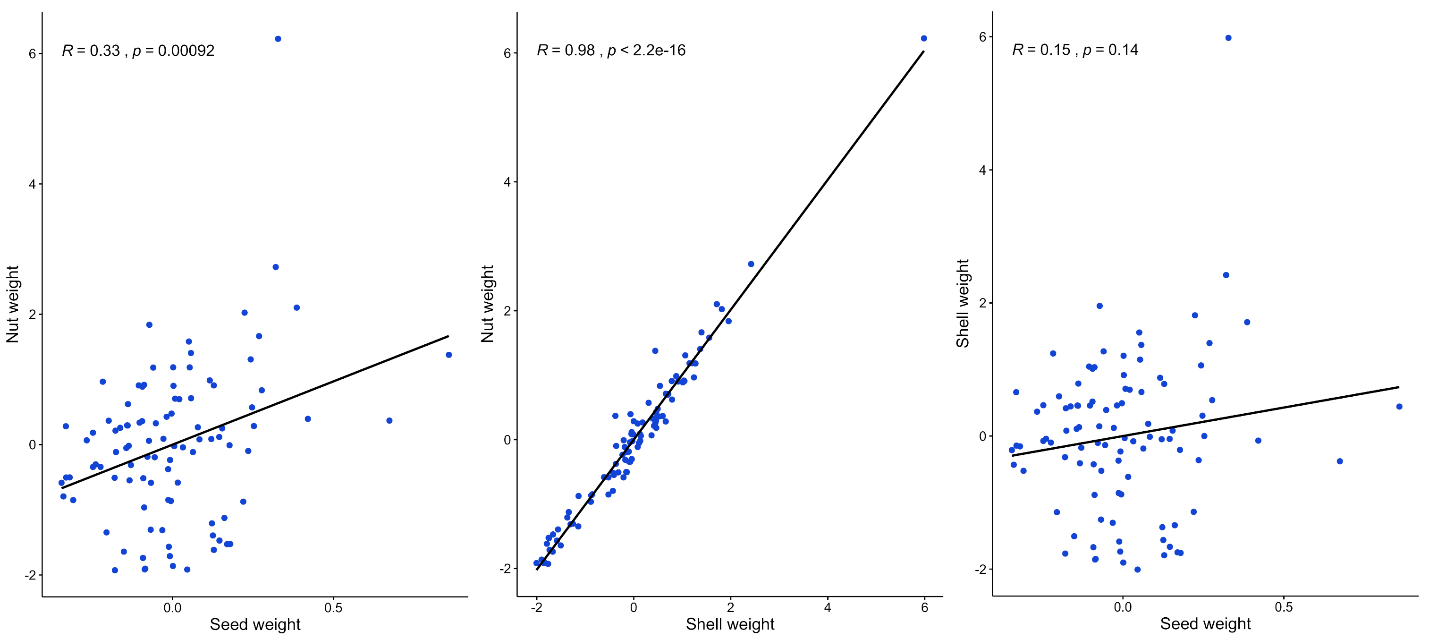


R^2^= 0.01, p = 0.1426

R^2^= 0.96, p = 2.2e-16

**a**

**b**

**c**
