# Supplementary material for: Almond diversity and homozygosity define structure, kinship, inbreeding, and linkage disequilibrium in cultivated germplasm, and reveal genomic associations with nut and seed weight
Source: Hortic Res. 2021 Jan 10;8:15. doi: 10.1038/s41438-020-00447-1 (PMC7797004; doi:10.1038/s41438-020-00447-1)
Supplement: Supplementary file 2 — Supplementary Tables [file 41438_2020_447_MOESM2_ESM.pdf]

Supporting Information Tables

Title: Diversity and homozygosity of almond define structure, kinship, inbreeding, and linkage disequilibrium in cultivated germplasm and reveal genomic associations with nut and seed weight

Authors: Stefano Pavan, Chiara Delvento, Rosa Mazzeo, Francesca Ricciardi, Pasquale Losciale, Liliana Gaeta, Nunzio D'Agostino, Francesca Taranto, Raquel Sánchez-Pérez, Luigi Ricciardi, Concetta Lotti.

Supporting information Table S1. Almond cultivars considered in this study, sampled from the ex situ collection of CREA-AA (Council for Agricultural Research and Analysis of Agricultural Economics, Section Agriculture and Environment, Bari, Italy) and CEBAS-CSICS (Centro de Edafología y Biología Aplicada del Segura, Consejo Superior de Investigaciones Científicas, Murcia, Spain). For each cultivar, information on the country of origin and, when available, the pedigree, is provided. Cultivars for which phenotypic data were collected are highlighted in bold.

Cultivar	Collection	Origin	Pedigree
**A grappolo**	CREA-AA	Italy	
Achaak	CEBAS-CSIC	Tunisia	
**Ai**	CREA-AA	France	
**Albanese**	CREA-AA	Italy	
Antoñeta	CEBAS-CSIC	Spain	Ferragnès x Tuono
**Antonio de Vito**	CREA-AA	Italy	
Antonio Pizzola	CREA-AA	Italy	
**Ardechoise**	CREA-AA	France	
Atocha	CEBAS-CSIC	Spain	
**Banchiere**	CREA-AA	Italy	
**Bartre**	CREA-AA	Spain	
Biancodda	CREA-AA	Italy	
**Bilarde**	CREA-AA	Italy	
**Burbank**	CREA-AA	U.S.	
Cacciola	CREA-AA	Italy	
Calcagno	CREA-AA	Italy	
**Caporusso**	CREA-AA	Italy	
**Caputo**	CREA-AA	Italy	
**Catalini**	CREA-AA	Italy	
Catuccia	CREA-AA	Italy	
**Catucedda**	CREA-AA	Italy	
**Cavaliera**	CREA-AA	Italy	
**Centopezze**	CREA-AA	Italy	
Chellastone	CEBAS-CSIC	Australia	
**Chino**	CREA-AA	Italy	
**Ciavea**	CREA-AA	Italy	
Cosimo di Bari	CREA-AA	Italy	
Crimsky	CREA-AA	Ukraine	
**Cristomorto**	CREA-AA	Italy	
**D'Aloia**	CREA-AA	Italy	
**Davey**	CREA-AA	U.S.	Nonpareil x Sans Faute
**Dehn**	CREA-AA	U.S.	
Del Cid	CEBAS-CSIC	Spain	
Del Lago	CREA-AA	Italy	
**Della Madonna di Molfetta**	CREA-AA	Italy	
**Della Madonna di San Giovanni Rotondo**	CREA-AA	Italy	
**Desmayo Largueta**	CREA-AA	Spain	
**Desmayo Rojo**	CREA-AA	Spain	
**Dorée**	CREA-AA	France	
**Drake**	CREA-AA	U.S.	
**Falsa Catuccia**	CREA-AA	Italy	
**Ferragnès**	CREA-AA	France	Cristomorto x Ai
**Ferrante**	CREA-AA	Italy	
Ficanera	CREA-AA	Italy	
**Ficarazza**	CREA-AA	Italy	
**Filippo Ceo**	CREA-AA	Italy	
**Flots**	CREA-AA	France	
**Fourcouronne**	CREA-AA	France	
**Fournat de Brézenaud**	CREA-AA	France	
Fragiulio	CREA-AA	Italy	
**Fragiulietta**	CREA-AA	Italy	
**Franciscudda**	CREA-AA	Italy	
**Galgano**	CREA-AA	Italy	
Garrigues	CEBAS-CSIC	Spain	
**Genco Laera**	CREA-AA	Italy	
Gioia	CREA-AA	Italy	
**Giunco di Cozze Alberobello**	CREA-AA	Italy	
Giunco di Cozze Ostuni	CREA-AA	Italy	
**Irene Lazolla**	CREA-AA	Italy	
Isa 200	CREA-AA	Italy	
IXL	CREA-AA	U.S.	
Kapareil	CREA-AA	U.S.	Nonpareil x Calif. 24-6
La parco forte	CREA-AA	Italy	
Lauranne	CEBAS-CSIC	Spain	
**Lorena Tribuzio**	CREA-AA	Italy	
M1-119	CEBAS-CSIC	Spagna	
**Malagueña**	CREA-AA	Spain	
**Mancina**	CREA-AA	Italy	
**Marchione**	CREA-AA	Italy	
**Marcona**	CREA-AA	Spain	
**Maria Carolina Tribuzio**	CREA-AA	Italy	
**Maria Tribuzio**	CREA-AA	Italy	
**Merced**	CREA-AA	U.S.	Nonpareil x Texas
Miagkoskorlupy	CREA-AA	Ukraine	
**Mincacetta**	CREA-AA	Italy	
**Mincone**	CREA-AA	Italy	
**Mollar de Taragona**	CREA-AA	Spain	
**Mollese di Canneto**	CREA-AA	Italy	
**Monaca**	CREA-AA	Italy	
Mono	CEBAS-CSIC	U.S.	
**Montrone**	CREA-AA	Italy	
Mosetta	CREA-AA	Italy	
**Ne plus ultra**	CREA-AA	U.S.	
Nessebre	CREA-AA	Ukraine	
**Nikitsky**	CREA-AA	Ukraine	
**Nocella**	CREA-AA	Italy	
Nonpareil	CREA-AA	U.S.	
**Occhio Rosso di Trani**	CREA-AA	Italy	
Pappamucco	CREA-AA	Italy	
**Peerlees**	CREA-AA	U.S.	
Peraleja	CEBAS-CSIC	Spain	
Pettolecchia	CREA-AA	Italy	
**Piangente**	CREA-AA	Italy	
**Picantili**	CREA-AA	Ukraine	
**Pignatidde tardiva**	CREA-AA	Italy	
**Piscalze**	CREA-AA	Italy	
**Pizzuta d'Avola**	CREA-AA	Italy	
**Primicerio**	CREA-AA	Italy	
**Primorsky**	CREA-AA	Ukraine	
**Pulita**	CREA-AA	Italy	
**Putignano**	CREA-AA	Italy	
R-1000	CEBAS-CSIC	France	
**Rabasse**	CREA-AA	France	
**Rachele**	CREA-AA	Italy	
**Rachele tenera**	CREA-AA	Italy	
**Rachelina**	CREA-AA	Italy	
Ramillete	CEBAS-CSIC	Spain	
Rana	CREA-AA	Italy	
**Rana gentile**	CREA-AA	Italy	
**Reale**	CREA-AA	Italy	
**Retsou**	CREA-AA	Greece	
**Ridenhome**	CREA-AA	U.S.	
**Riviello**	CREA-AA	Italy	
**Rossa**	CREA-AA	Italy	
S3067	CEBAS-CSIC	Spain	
**Santeramo**	CREA-AA	Italy	
**Santoro**	CREA-AA	Italy	
SAS 15	CREA-AA	Italy	
**Scorza verde**	CREA-AA	Italy	
Selvatico	CREA-AA	Italy	
**Senz'arte**	CREA-AA	Italy	
Spina	CREA-AA	Italy	
**Sultana**	CREA-AA	France	
**Symmetrike**	CREA-AA	Greece	
Supernova	CREA-AA	Italy	
**Tardy nonpareil**	CREA-AA	U.S.	
Tedone	CREA-AA	Italy	
**Tenente**	CREA-AA	Italy	
**Texas**	CREA-AA	U.S.	
Tioga	CEBAS-CSIC	U.S.	
Titan	CEBAS-CSIC	U.S.	
**Tondina**	CREA-AA	Italy	
**Tournefort**	CREA-AA	France	
Tranella	CREA-AA	Italy	
**Tribuzio**	CREA-AA	Italy	
Troito	CREA-AA	Italy	
Tuono	CREA-AA	Italy	
**Vesta**	CREA-AA	U.S.	
**Viscardia**	CREA-AA	Italy	
Vitantonio	CREA-AA	Italy	
Vivot	CEBAS-CSIC	Spain	
**Vuoi o non vuoi**	CREA-AA	Italy	
Wawona	CEBAS-CSIC	U.S.	
**Yaltinsky**	CREA-AA	Ukraine	
**Zanzanidde**	CREA-AA	Italy	
**Zia Comara**	CREA-AA	Italy	
**Zin zin**	CREA-AA	Italy	
Zio Gaetano	CREA-AA	Italy	
Zio Pietro	CREA-AA	Italy	

Supporting information Table S2. Composition of the clonal groups (CGs) identified in this study. The cultivars displaying the highest call rate, chosen to represent each CG, are highlighted in bold.

Genotype	Origin	Group
Troito	Italy	1
Supernova	Italy	
Tuono	Italy	
Miagkoskorlupy	Ukraine	
**Chino**	Italy	
Pettolecchia	Italy	2
Gioia	Italy	
**Marchione**	Italy	
**Fragiulietta**	Italy	3
Fragiulio	Italy	
**Monaca**	Italy	4
La parco forte	Italy	
IXL	U.S.	5
**Ridenhome**	U.S.	
**Rana gentile**	Italy	6
Rana	Italy	
Pappamucco	Italy	7
**Banchiere**	Italy	
Cosimo di Bari	Italy	8
**Pignatidde tardiva**	Italy	
**Cristomorto**	Italy	9
Del Lago	Italy	
Catuccia	Italy	10
**Catucedda**	Italy	
Crimsky	Ukraine	11
**Peerlees**	U.S.	

Supporting information Table S3. Identity by descent (IBD) matrix calculated on the almond cultivars considered in this study. Clonal groups are represented by the cultivar displaying the highest SNP call rate, as indicated in Table S2.

Genotype	Selvatico	Fragiulletta (CG3)	Primicerio	Davey	Zio Gaetano	Fourcouronne	Yaltinsky	A grappolo	Sultana	Tardy nonpareil	Biancodda	Cristomorto (CG8)	Ficarazza	Lorena Tributo	Mancina	Kapareil	Picantili	Rabasse	Pulita	Albanese	Rana gentile (CG6)	Vitantonio	Filippo Ceo
Selvatico	1	0	0	0	0	0	0	0	0	0	0	0	0	0	0	0	0	0	0	0	0	0	0
Fragiulletta (CG3)	0	1	0	0	0	0	0	0	0	0	0	0	0	0	0	0	0	0	0	0	0	0	0
Primicerio	0	0	1	0	0	0	0	0	0	0	0	0	0	0	0	0	0	0	0	0	0	0	0
Davey	0	0	0	1	0	0	0	0	0	0	0	0	0	0	0	0	0	0	0	0	0	0	0
Zio Gaetano	0	0	0	0	1	0	0	0	0	0	0	0	0	0	0	0	0	0	0	0	0	0	0
Fourcouronne	0	0	0	0	0	1	0	0	0	0	0	0	0	0	0	0	0	0	0	0	0	0	0
Yaltinsky	0	0	0	0	0	0	1	0	0	0	0	0	0	0	0	0	0	0	0	0	0	0	0
A grappolo	0	0	0	0	0	0	0	1	0	0	0	0	0	0	0	0	0	0	0	0	0	0	0
Sultana	0	0	0	0	0	0	0	0	1	0	0	0	0	0	0	0	0	0	0	0	0	0	0
Tardy nonpareil	0	0	0	0	0	0	0	0	0	1	0	0	0	0	0	0	0	0	0	0	0	0	0
Biancodda	0	0	0	0	0	0	0	0	0	0	1	0	0	0	0	0	0	0	0	0	0	0	0
Cristomorto (CG8)	0	0	0	0	0	0	0	0	0	0	0	1	0.1494	0	0	0	0.2664	0	0	0	0	0	0
Ficarazza	0	0	0	0	0	0	0	0	0	0	0	0.1494	1	0	0	0	0.2296	0	0	0	0	0	0
Lorena Tributo	0	0	0	0	0	0	0	0	0	0	0	0	0	1	0	0	0	0	0	0	0	0	0
Mancina	0	0	0	0	0	0	0	0	0	0	0	0	0	0	1	0	0	0	0	0	0	0	0
Kapareil	0	0	0	0	0	0	0	0	0	0	0	0	0	0	0	1	0	0	0	0	0	0	0
Picantili	0	0	0	0	0	0	0	0	0	0	0	0.2664	0.2296	0	0	0	1	0.2143	0	0	0	0	0
Rabasse	0	0	0	0	0	0	0	0	0	0	0	0	0	0	0	0	0.2142	1	0	0	0	0	0
Pulita	0	0	0	0	0	0	0	0	0	0	0	0	0	0	0	0	0	0	1	0	0	0	0
Albanese	0	0	0	0	0	0	0	0	0	0	0	0	0	0	0	0	0	0	0	1	0	0	0
Rana gentile (CG6)	0	0	0	0	0	0	0	0	0	0	0	0	0	0	0	0	0	0	0	0	1	0	0
Vitantonio	0	0	0	0	0	0	0	0	0	0	0	0	0	0	0	0	0	0	0	0	0	1	0
Filippo Ceo	0	0	0	0	0	0	0	0	0	0	0	0	0	0	0	0	0	0	0	0	0	0	1
Burbank	0	0	0	0	0	0	0	0	0	0	0	0	0	0	0	0	0	0	0	0	0	0	0
Merced	0	0	0	0	0	0	0	0	0	0	0	0	0	0	0	0	0	0	0	0	0	0	0
Scorza verde	0	0	0	0	0	0	0	0	0	0	0	0	0	0	0	0	0	0	0	0	0	0	0
Piangente	0	0	0	0	0	0	0	0	0	0	0	0	0	0	0	0	0	0	0	0	0	0	0
Giunco Ostuni	0	0	0	0	0	0	0	0	0	0	0	0	0	0	0	0	0	0	0	0	0	0	0

Table S4. Mean r^2 for markers within individual chromosomes.

Chromosome	Mean r^2
1	0.142
2	0.147
3	0.136
4	0.145
5	0.141
6	0.132
7	0.143
8	0.144

Table S5. HM results for nut, shell and seed weight. The table reports genomic coordinates, p-value, FDR-corrected p-value and annotated genes of trimmed clusters of ROHs, obtained from original clusters of ROHs with length > 100 Kb to group cultivars with highly overlapping homozygous regions.

Nut/shell weight

ROH name	Chromosome	Start position	End position	Length	#SNP	$-log_{10}$ (p-value)		FDR p-value		Gene name	Gene function
						Nut weight	Shell weight	Nut weight	Shell weight		
ROH_1_11260515	1	11260515	11331072	70557	39	4.738583419	4.665867493	0.008589665	0.010155285	Prudu_001271_v1.0	Heavy metal transport/detoxification superfamily protein
										Prudu_001272_v1.0	Basic helix-loop-helix DNA-binding superfamily protein
										Prudu_001273_v1.0	Dual specificity protein phosphatase 1
										Prudu_001274_v1.0	Hypothetical protein
										Prudu_001275_v1.0	NOD26-like intrinsic protein 7
										Prudu_001277_v1.0	Hypothetical protein
										Prudu_001276_v1.0	Aluminium induced protein with YGL and LRDR motifs
										Prudu_001277_v1.0	Hypothetical protein
ROH_2_16414730	2	16414730	16460691	45961	8	5.168428842	5.404225561	0.006384998	0.003709916	Prudu_006731_v1.0	PLAC8 family protein
										Prudu_006732_v1.0	PLAC8 family protein
										Prudu_006733_v1.0	Hypothetical protein
										Prudu_006734_v1.0	Hypothetical protein
										Prudu_006735_v1.0	Quinoprotein amine dehydrogenase, beta chain-like
										Prudu_006736_v1.0	Ferric reduction oxidase 2
										Prudu_006737_v1.0	Protein of unknown function D
ROH_7_11701812	7	11701812	11769914	68102	16	3.911390436	4.427086155	0.03846608	0.011732274	Prudu_019716_v1.0	Nucleotide-diphospho-sugar transferases superfamily protein
										Prudu_019717_v1.0	Haloacid dehalogenase-like hydrolase superfamily protein
										Prudu_019718_v1.0	Haloacid dehalogenase-like hydrolase superfamily protein
										Prudu_019719_v1.0	AT-hook motif nuclear-localized protein 1
										Prudu_019720_v1.0	Cytochrome P450, family 76, subfamily C, polypeptide 4
										Prudu_019721_v1.0	Cytochrome P450, family 76, subfamily G, polypeptide 1
										Prudu_019722_v1.0	KDO transferase A

Seed weight

ROH name	Chromosome	Start position	End position	Length	#SNP	$-log_{10}$ (p-value)	FDR p-value	Gene name	Gene function
ROH_1_26367663	1	26367663	26508468	140805	39	4.066560244	0.080728964	Prudu_002814_v1.0	MATE efflux family protein
								Prudu_002815_v1.0	MATE efflux family protein
								Prudu_002816_v1.0	ASF1 like histone chaperone
								Prudu_002817_v1.0	ATP synthase alpha/beta family protein
								Prudu_002818_v1.0	Hypothetical protein
								Prudu_002819_v1.0	Hypothetical protein
								Prudu_002820_v1.0	Hypothetical protein
								Prudu_002821_v1.0	Hypothetical protein
								Prudu_002822_v1.0	Hypothetical protein
								Prudu_002823_v1.0	Endosomal targeting BRO1-like domain-containing protein
								Prudu_002824_v1.0	Hypothetical protein
								Prudu_002825_v1.0	Hypothetical protein
								Prudu_002826_v1.0	S-adenosyl-L-methionine-dependent methyltransferases superfamily protein
								Prudu_002827_v1.0	Hypothetical protein
								Prudu_002828_v1.0	Hypothetical protein
								Prudu_002829_v1.0	Cytochrome P450, family 716, subfamily A, polypeptide 1
								Prudu_002830_v1.0	Cytochrome P450, family 716, subfamily A, polypeptide 1
								Prudu_002831_v1.0	S-adenosyl-L-methionine-dependent methyltransferases superfamily protein
								Prudu_002832_v1.0	S-adenosyl-L-methionine-dependent methyltransferases superfamily protein
								Prudu_002833_v1.0	Tetratricopeptide repeat-like superfamily protein
								Prudu_002834_v1.0	GDSL-motif lipase/hydrolase 6
								Prudu_002835_v1.0	Transcription regulators
								Prudu_002836_v1.0	Quiescin-sulfhydryl oxidase 2
								Prudu_002837_v1.0	RING/FYVE/PHD zinc finger superfamily protein
								Prudu_002838_v1.0	Hypothetical protein
								Prudu_002839_v1.0	Ribose-5-phosphate isomerase 2
								Prudu_002840_v1.0	ABC-2 type transporter family protein
								Prudu_002841_v1.0	RNA polymerase II transcription elongation factor
								Prudu_002842_v1.0	Core-2/I-branching beta-1,6-N-acetylglucosaminyltransferase family protein
								Prudu_002843_v1.0	Hypothetical protein
								Prudu_002844_v1.0	Heavy metal transport/detoxification superfamily protein
ROH_S6_20767156	6	20767156	20833595	66439	11	3.848461767	0.066695704	Prudu.06G223800	Heavy metal atpase 1
								Prudu.06G223900	FAD-dependent oxidoreductase family protein
								Prudu.06G224000	Hypothetical protein
								Prudu.06G224100	Leucine-rich repeat protein kinase family protein
								Prudu.06G224200	End binding protein 1C
								Prudu.06G224300	ADP-ribosylation factor A1F
								Prudu.06G224400	CYCLIN D3
								Prudu.06G224500	Hypothetical protein
								Prudu.06G224600	PTEN 2

Supporting information Table S6. GWAS results. For each SNP, information is provided on genomic coordinates, p-value and FDR-corrected p-value. The table also reports the gene in which the SNP resides or, in case of intergenic localization, the two genes flanking the SNP.

Nut weight						
**Marker name**	**Chromosome**	**Position**	**- log10 (p-value)**	**FDR p-value**	**Gene name**	**Gene function**
S1_7504639	1	7504639	5.605232724	0.019259	Prudu_000837_v1.0	NAD(P)-binding Rossmann-fold superfamily protein
S1_30936643	1	30936643	11.05395391	0.000001	Prudu_003449_v1.0; Prudu_003450_v1.0	Eukaryotic aspartyl protease family protein; Hypothetical protein
S1_43618394	1	43618394	4.674920027	0.056242	Prudu_005021_v1.0	Hypothetical protein
S2_354742	2	354742	5.263842635	0.029837	Prudu_005063_v1.0	DNA-binding bromodomain-containing protein
S2_354762	2	354762	5.263842635	0.028179	Prudu_005063_v1.0	DNA-binding bromodomain-containing protein
S2_5131274	2	5131274	5.176695031	0.029521	Prudu_005565_v1.0; Prudu_005566_v1.0	Light-mediated development protein 1 / deetiolated1; Hypothetical protein
S2_5131329	2	5131329	5.176695031	0.028179	Prudu_005565_v1.0; Prudu_005566_v1.0	Light-mediated development protein 1 / deetiolated1; Hypothetical protein
S2_5131345	2	5131345	4.491711766	0.062531	Prudu_005565_v1.0; Prudu_005566_v1.0	Light-mediated development protein 1 / deetiolated1; Hypothetical protein
S2_5131375	2	5131375	4.491711766	0.061255	Prudu_005565_v1.0; Prudu_005566_v1.0	Light-mediated development protein 1 / deetiolated1; Hypothetical protein
S2_5678957	2	5678957	5.860832828	0.018328	Prudu_005622_v1.0	ABA-responsive element binding protein 3
S2_5678998	2	5678998	5.793766928	0.018715	Prudu_005622_v1.0	ABA-responsive element binding protein 3
S2_5956213	2	5956213	5.687069407	0.017401	Prudu_005641_v1.0; Prudu_005642_v1.0	Transposable element gene; Hypothetical protein
S2_6108424	2	6108424	5.155748778	0.028286	Prudu_005661_v1.0; Prudu_005662_v1.0	Ethylene insensitive 3 family protein; Hypothetical protein
S2_18293846	2	18293846	4.433623644	0.067275	Prudu_006966_v1.0	hypothetical protein
S2_19461071	2	19461071	5.219595912	0.028082	Prudu_007119_v1.0; Prudu_007120_v1.0	Protein of unknown function D; Binding
S2_19949432	2	19949432	4.647528253	0.055174	Prudu_007177_v1.0	3-ketoacyl-CoA synthase 19
S2_20396061	2	20396061	4.662914268	0.054693	Prudu_007245_v1.0; Prudu_007246_v1.0	Heat shock protein 70; 3-oxo-5-alpha-steroid 4-dehydrogenase family protein
S2_24520281	2	24520281	4.641555661	0.054504	Prudu_007887_v1.0	Scramblase-related
S2_24572872	2	24572872	4.347103564	0.079007	Prudu_007894_v1.0	Exportin 1A
S3_9870445	3	9870445	4.530338864	0.061023	Prudu_009079_v1.0; Prudu_009080_v1.0;	TRICHOME BIREFRINGENCE-LIKE 39; Hypothetical protein
S3_18482351	3	18482351	4.8737811	0.044474	Prudu_009972_v1.0	Diacylglycerol kinase 5
S3_18772445	3	18772445	4.668208499	0.055531	Prudu_010006_v1.0; Prudu_010007_v1.0;	Peroxisomal membrane 22 kDa Mpv17/PMP22 family protein; Protein phosphatase 2C family protein
S3_23053252	3	23053252	5.034430991	0.035843	Prudu_010621_v1.0	ARM repeat superfamily protein
S4_8267	4	8267	5.551998626	0.020096	Prudu_010622_v1.0	Family of unknown function (DUF566)
S4_216646	4	216646	4.494000949	0.064907	Prudu_010662_v1.0	Nudix hydrolase homolog 2
S4_6118251	4	6118251	6.044758394	0.016800	Prudu_011538_v1.0; Prudu_011539_v1.0	NAC domain containing protein 47; Hypothetical protein
S4_6118274	4	6118274	6.044758394	0.014000	Prudu_011538_v1.0; Prudu_011539_v1.0	NAC domain containing protein 47; Hypothetical protein
S4_13996635	4	13996635	5.30745925	0.028673	Prudu_012488_v1.0	Ethylene response factor 7
S4_20427524	4	20427524	4.57077903	0.059569	Prudu_013094_v1.0	Prefoldin chaperone subunit family protein
S5_3591231	5	3591231	4.493229974	0.063638	Prudu_013568_v1.0	Glutaredoxin family protein
S5_5517838	5	5517838	4.33593234	0.079565	Prudu_013764_v1.0	Restriction endonuclease, type II-like superfamily protein
S5_5517863	5	5517863	4.33593234	0.078118	Prudu_013764_v1.0	Restriction endonuclease, type II-like superfamily protein
S5_5850001	5	5850001	5.523866413	0.019909	Prudu_013793_v1.0; Prudu_013794_v1.0	Hypothetical protein; Transposable element gene
S5_5995957	5	5995957	5.757140254	0.018099	Prudu_013808_v1.0	Hypothetical protein
S6_17341954	6	17341954	4.46686859	0.063564	Prudu_017135_v1.0; Prudu_017136_v1.0	DNAse I-like superfamily protein; Protein of unknown function D
S6_17901146	6	17901146	4.55523894	0.058933	Prudu_017185_v1.0; Prudu_017186_v1.0	Cyclin-dependent kinase C; Hypothetical protein
S6_19133098	6	19133098	4.714592105	0.056144	Prudu_017312_v1.0; Prudu_017313_v1.0	UDP-Glycosyltransferase superfamily protein; EF-TU receptor
S6_19133126	6	19133126	4.714592105	0.054443	Prudu_017312_v1.0; Prudu_017313_v1.0	UDP-Glycosyltransferase superfamily protein; EF-TU receptor
S6_24920494	6	24920494	4.561122407	0.059492	Prudu_018019_v1.0	Malectin/receptor-like protein kinase family protein
S6_25129258	6	25129258	4.243183298	0.093323	Prudu_018053_v1.0	Leucine-rich repeat protein kinase family protein
S6_25403733	6	25403733	4.273852348	0.088513	Prudu_018088_v1.0	MuDR family transposase
S7_8761615	7	8761615	5.33903461	0.028439	Prudu_019377_v1.0	Nse4, component of Smc5/6 DNA repair complex
S7_8761617	7	8761617	4.967378364	0.037180	Prudu_019377_v1.0	Nse4, component of Smc5/6 DNA repair complex
S7_16041360	7	16041360	4.81870369	0.048747	Prudu_020294_v1.0	ATP binding microtubule motor family protein
S7_16150885	7	16150885	5.258819977	0.027007	Prudu_020307_v1.0	Auxin response factor 19
S7_18587295	7	18587295	5.026766585	0.035022	Prudu_020680_v1.0	Mitochondrial substrate carrier family protein
S7_18587297	7	18587297	5.026766585	0.033675	Prudu_020680_v1.0	Mitochondrial substrate carrier family protein
S7_18635252	7	18635252	6.341744231	0.010598	Prudu_020694_v1.0	Alkenal reductase
S8_3891561	8	3891561	4.374048185	0.075683	Prudu_021383_v1.0	MA3 domain-containing protein
S8_8835196	8	8835196	4.688720244	0.056085	Prudu_021821_v1.0	Vacuolar proton ATPase A3
S8_12995325	8	12995325	4.757604781	0.054240	Prudu_022298_v1.0	F-box/RNI-like superfamily protein
S8_13031235	8	13031235	4.621345068	0.055673	Prudu_022300_v1.0	Protein kinase superfamily protein
S8_13053739	8	13053739	4.726427345	0.056397	Prudu_022303_v1.0	Calmodulin-binding family protein
S8_13649088	8	13649088	4.600740998	0.056954	Prudu_022389_v1.0	Disease resistance-responsive dirigent-like family protein
S8_14117420	8	14117420	5.745769916	0.016722	Prudu_022449_v1.0	Hypothetical protein
S8_16914707	8	16914707	6.717934899	0.008914	Prudu_022861_v1.0	Breast cancer associated RING 1
S8_16914743	8	16914743	6.717934899	0.005943	Prudu_022861_v1.0	Breast cancer associated RING 1

Shell weight						
**Marker name**	**Chromosome**	**Position**	**- log10 (p-value)**	**FDR p-value**	**Gene name**	**Gene function**
S1_7504639	1	7504639	5.809620361	0.0144353	Prudu_000837_v1.0	NAD(P)-binding Rossmann-fold superfamily protein
S1_21663148	1	21663148	4.146133348	0.0852746	Prudu_002253_v1.0; Prudu_002254_v1.0	Plant protein of unknown function D; NSP-interacting kinase 3
S1_21663219	1	21663219	4.146133348	0.0841952	Prudu_002253_v1.0; Prudu_002254_v1.0	Plant protein of unknown function D; NSP-interacting kinase 3
S1_21663244	1	21663244	4.146133348	0.0831427	Prudu_002253_v1.0; Prudu_002254_v1.0	Plant protein of unknown function D; NSP-interacting kinase 3
S1_27205051	1	27205051	4.464589008	0.0726116	Prudu_002930_v1.0	Hypothetical protein
S1_30936643	1	30936643	11.82220767	0.0000001	Prudu_003449_v1.0; Prudu_003450_v1.0	Eukaryotic aspartyl protease family protein; Hypothetical protein
S1_35460840	1	35460840	4.109190102	0.0883169	Prudu_004029_v1.0; Prudu_004030_v1.0	Pyrimidine 2; Transposable element genetransposable element gene
S1_39316449	1	39316449	4.269667877	0.0848258	Prudu_004513_v1.0	Hypothetical protein
S1_43618394	1	43618394	5.363929586	0.0223793	Prudu_005021_v1.0	Hypothetical protein
S2_354742	2	354742	5.225668102	0.0263730	Prudu_005063_v1.0	DNA-binding bromodomain-containing protein
S2_354762	2	354762	5.225668102	0.0251743	Prudu_005063_v1.0	DNA-binding bromodomain-containing protein
S2_5131274	2	5131274	4.863800715	0.0439389	Prudu_005565_v1.0; Prudu_005566_v1.0	Light-mediated development protein 1 / deetiolated1; Hypothetical protein
S2_5131329	2	5131329	4.863800715	0.0424742	Prudu_005565_v1.0; Prudu_005566_v1.0	Light-mediated development protein 1 / deetiolated1; Hypothetical protein
S2_5131345	2	5131345	4.199546543	0.0828405	Prudu_005565_v1.0; Prudu_005566_v1.0	Light-mediated development protein 1 / deetiolated1; Hypothetical protein
S2_5131375	2	5131375	4.199546543	0.0816899	Prudu_005565_v1.0; Prudu_005566_v1.0	Light-mediated development protein 1 / deetiolated1; Hypothetical protein
S2_5678957	2	5678957	5.761240784	0.0146695	Prudu_005622_v1.0	ABA-responsive element binding protein 3
S2_5678998	2	5678998	5.727532754	0.0145323	Prudu_005622_v1.0	ABA-responsive element binding protein 3
S2_5956213	2	5956213	5.326380085	0.0231161	Prudu_005641_v1.0; Prudu_005642_v1.0	Transposable element gene; Hypothetical protein
S2_6108424	2	6108424	5.078248939	0.0311070	Prudu_005661_v1.0; Prudu_005662_v1.0	Ethylene insensitive 3 family protein; Hypothetical protein
S2_12269572	2	12269572	4.444835327	0.0743019	Prudu_006265_v1.0; Prudu_006266_v1.0	Duplicated homeodomain-like superfamily protein; purine permease 11
S2_16706061	2	16706061	4.351309235	0.0740543	Prudu_006774_v1.0	Alpha/beta-Hydrolases superfamily protein
S2_19461071	2	19461071	5.515571972	0.0177564	Prudu_007119_v1.0; Prudu_007120_v1.0	Protein of unknown function D; Binding
S2_19949432	2	19949432	4.855488393	0.0418984	Prudu_007177_v1.0	3-ketoacyl-CoA synthase 19
S2_20364878	2	20364878	4.398618127	0.0774798	Prudu_007238_v1.0	Heat shock protein 70
S2_20364881	2	20364881	4.398618127	0.0758985	Prudu_007238_v1.0	Heat shock protein 70
S2_20369938	2	20369938	4.398618127	0.0743806	Prudu_007239_v1.0	Mitochondrial HSO70 2
S2_20369952	2	20369952	4.398618127	0.0729221	Prudu_007239_v1.0	Mitochondrial HSO70 2
S2_20369959	2	20369959	4.398618127	0.0715198	Prudu_007239_v1.0	Mitochondrial HSO70 2
S2_20378329	2	20378329	4.142540842	0.0827984	Prudu_007243_v1.0	Hypothetical protein
S2_20396061	2	20396061	4.635068099	0.0583147	Prudu_007245_v1.0; Prudu_007246_v1.0	Heat shock protein 70; 3-oxo-5-alpha-steroid 4-dehydrogenase family protein
S2_23011819	2	23011819	4.090907926	0.0899209	Prudu_007630_v1.0; Prudu_007631_v1.0	Ubiquitin carboxyl-terminal hydrolase family protein; XB3 ortholog 3 in Arabidopsis thaliana
S2_24520281	2	24520281	4.504809281	0.0677283	Prudu_007887_v1.0	Scramblase-related
S2_24572872	2	24572872	5.122261261	0.0292803	Prudu_007894_v1.0	Exportin 1A
S2_24695329	2	24695329	4.267382748	0.0838520	Prudu_007918_v1.0	RNA-binding family protein with RRM/RBD/RNP motifs
S2_24695332	2	24695332	4.267382748	0.0824774	Prudu_007918_v1.0	RNA-binding family protein with RRM/RBD/RNP motifs
S2_24695334	2	24695334	4.267382748	0.0811471	Prudu_007918_v1.0	RNA-binding family protein with RRM/RBD/RNP motifs
S3_9870445	3	9870445	4.717371195	0.0510045	Prudu_009079_v1.0; Prudu_009080_v1.0;	TRICHOME BIREFRINGENCE-LIKE 39; Hypothetical protein
S3_18482351	3	18482351	4.232672775	0.0813392	Prudu_009972_v1.0	Diacylglycerol kinase 5
S3_18772445	3	18772445	3.985612877	0.1046278	Prudu_010006_v1.0; Prudu_010007_v1.0;	Peroxisomal membrane 22 kDa Mpv17/PMP22 family protein; Protein phosphatase 2C family protein
S3_20640429	3	20640429	4.362919817	0.0747711	Prudu_010259_v1.0; Prudu_010260_v1.0	hypothetical protein; Pentatricopeptide repeat superfamily protein
S3_22621775	3	22621775	4.151487844	0.0875989	Prudu_010554_v1.0; Prudu_010555_v1.0	Major facilitator superfamily protein; K-box region and MADS-box transcription factor family protein
S3_22621789	3	22621789	4.151487844	0.0864463	Prudu_010554_v1.0; Prudu_010555_v1.0	Major facilitator superfamily protein; K-box region and MADS-box transcription factor family protein
S3_23053252	3	23053252	4.353709828	0.0749851	Prudu_010621_v1.0	ARM repeat superfamily protein
S4_8267	4	8267	6.02157993	0.0126581	Prudu_010622_v1.0	Family of unknown function (DUF566)
S4_45072	4	45072	4.610846934	0.0600367	Prudu_010627_v1.0; Prudu_010628_v1.0	Myb-like HTH transcriptional regulator family protein; Homeodomain-like superfamily protein
S4_167697	4	167697	4.610846934	0.0584973	Prudu_010650_v1.0	Hypothetical protein
S4_168317	4	168317	4.610846934	0.0570349	Prudu_010650_v1.0	Hypothetical protein
S4_216646	4	216646	4.97493135	0.0352337	Prudu_010662_v1.0	Nudix hydrolase homolog 2
S4_2707351	4	2707351	4.150297681	0.0855577	Prudu_011047_v1.0	Seven transmembrane MLO family protein
S4_3708834	4	3708834	4.254678831	0.0796995	Prudu_011198_v1.0	AWPM-19-like family protein
S4_3708936	4	3708936	4.254678831	0.0784919	Prudu_011198_v1.0	AWPM-19-like family protein
S4_6118251	4	6118251	5.697806509	0.0133387	Prudu_011538_v1.0; Prudu_011539_v1.0	NAC domain containing protein 47; Hypothetical protein
S4_6118274	4	6118274	5.697806509	0.0124494	Prudu_011538_v1.0; Prudu_011539_v1.0	NAC domain containing protein 47; Hpothetical protein
S4_13996635	4	13996635	6.312659004	0.0113326	Prudu_012488_v1.0	Ethylene response factor 7
S4_20427524	4	20427524	4.341790275	0.0743674	Prudu_013094_v1.0	Prefoldin chaperone subunit family protein
S5_3591231	5	3591231	3.930993519	0.1039597	Prudu_013568_v1.0	Glutaredoxin family protein
S5_5517838	5	5517838	4.159282843	0.0883979	Prudu_013764_v1.0	Restriction endonuclease, type II-like superfamily protein
S5_5517863	5	5517863	4.159282843	0.0872033	Prudu_013764_v1.0	Restriction endonuclease, type II-like superfamily protein
S5_5850001	5	5850001	5.854989063	0.0144483	Prudu_013793_v1.0; Prudu_013794_v1.0	Hypothetical protein; Transposable element gene
S5_5995957	5	5995957	6.104893448	0.0121900	Prudu_013808_v1.0	Hypothetical protein
S5_6247676	5	6247676	4.31515977	0.0777070	Prudu_013837_v1.0	Photosystem II reaction center protein B
S6_8270805	6	8270805	4.061948958	0.0938859	Prudu_016370_v1.0	PHD finger family protein
S6_17341954	6	17341954	4.203374211	0.0832866	Prudu_017135_v1.0; Prudu_017136_v1.0	DNAse I-like superfamily protein; Protein of unknown function D
S6_17901146	6	17901146	4.256572602	0.0805926	Prudu_017185_v1.0; Prudu_017186_v1.0	Cyclin-dependent kinase C; Hypothetical protein
S6_19133098	6	19133098	4.440176645	0.0734705	Prudu_017312_v1.0; Prudu_017313_v1.0	UDP-Glycosyltransferase superfamily protein; EF-TU receptor
S6_19133126	6	19133126	4.440176645	0.0719073	Prudu_017312_v1.0; Prudu_017313_v1.0	UDP-Glycosyltransferase superfamily protein; EF-TU receptor
S6_24920494	6	24920494	4.72048296	0.0521298	Prudu_018019_v1.0	Malectin/receptor-like protein kinase family protein
S6_25129258	6	25129258	4.263826389	0.0805157	Prudu_018053_v1.0	Leucine-rich repeat protein kinase family protein
S6_25403733	6	25403733	3.943009619	0.1030863	Prudu_018088_v1.0	MuDR family transposase
S7_1537228	7	1537228	4.778965459	0.0469427	Prudu_018753_v1.0	Floral homeotic protein HUA1
S7_8761615	7	8761615	5.923794054	0.0138728	Prudu_019377_v1.0	Nse4, component of Smc5/6 DNA repair complex
S7_8761617	7	8761617	5.468613734	0.0186202	Prudu_019377_v1.0	Nse4, component of Smc5/6 DNA repair complex
S7_9831726	7	9831726	4.231677005	0.0803270	Prudu_019516_v1.0; Prudu_019517_v1.0	Pentatricopeptide repeat superfamily protein; DNAJ heat shock N-terminal domain-containing protein
S7_10088585	7	10088585	4.203558061	0.0844579	Prudu_019538_v1.0; Prudu_019539_v1.0	Aluminium activated malate transporter family protein; Hypothetical protein
S7_16041360	7	16041360	4.780793043	0.0482064	Prudu_020294_v1.0	ATP binding microtubule motor family protein
S7_16150885	7	16150885	5.219368855	0.0244315	Prudu_020307_v1.0	Auxin response factor 19
S7_18587295	7	18587295	4.979305576	0.0375637	Prudu_020680_v1.0	Mitochondrial substrate carrier family protein
S7_18587297	7	18587297	4.979305576	0.0361725	Prudu_020680_v1.0	Mitochondrial substrate carrier family protein
S7_18635252	7	18635252	5.720281179	0.0136403	Prudu_020694_v1.0	Alkenal reductase
S8_3891561	8	3891561	4.379182004	0.0733820	Prudu_021383_v1.0	MA3 domain-containing protein
S8_4168319	8	4168319	4.07106106	0.0930181	Prudu_021419_v1.0	Laccase/Diphenol oxidase family protein
S8_8835196	8	8835196	4.104105668	0.0882803	Prudu_021821_v1.0	Vacuolar proton ATPase A3
S8_12995325	8	12995325	4.66140454	0.0564080	Prudu_022298_v1.0	F-box/RNI-like superfamily protein
S8_13031235	8	13031235	4.505483705	0.0692332	Prudu_022300_v1.0	Protein kinase superfamily protein
S8_13053739	8	13053739	4.546798616	0.0644860	Prudu_022303_v1.0	Calmodulin-binding family protein
S8_13649088	8	13649088	5.245735651	0.0264413	Prudu_022389_v1.0	Disease resistance-responsive dirigent-like family protein
S8_14117420	8	14117420	6.157431333	0.0129612	Prudu_022449_v1.0	Hypothetical protein
S8_16914707	8	16914707	6.916781323	0.0056394	Prudu_022861_v1.0	Breast cancer associated RING 1
S8_16914743	8	16914743	6.916781323	0.0037596	Prudu_022861_v1.0	Breast cancer associated RING 1

Seed weight						
**Marker name**	**Chromosome**	**Position**	**- log10 (p-value)**	**FDR p-value**	**Gene name**	**Gene function**
S1_2496687	1	2496687	6.19	0.060724462	Prudu_000307_v1.0	Pathogenesis-related thaumatin superfamily protein
